# Development of a prognostic risk model of uveal melanoma based on N7-methylguanosine-related regulators

**DOI:** 10.1186/s41065-024-00324-0

**Published:** 2024-07-10

**Authors:** Pingfan Wu, Qian Zhang, Peng Zhong, Li Chai, Qiong Luo, Chengyou Jia

**Affiliations:** 1 Department of Nuclear Medicine, Shanghai Tenth People’s Hospital, Tongji University School of Medicine, Shanghai, 200072 China; 2 Institute of Nuclear Medicine, Tongji University School of Medicine, Shanghai, 200072 China; 3 Department of Plastic and Aesthetic Surgery, The Second Affiliated Hospital of Soochow University, Suzhou, 215004 China

**Keywords:** Uveal melanoma, N7-methylguanosine, Prognostic Risk Model, Immune infiltration, Immune checkpoint

## Abstract

**Background:**

Uveal melanoma (UVM) stands as the predominant type of primary intraocular malignancy among adults. The clinical significance of N7-methylguanosine (m7G), a prevalent RNA modifications, in UVM remains unclear.

**Methods:**

Primary information from 80 UVM patients were analyzed as the training set, incorporating clinical information, mutation annotations and mRNA expression obtained from The Cancer Genome Atlas (TCGA) website. The validation set was carried out using Gene Expression Omnibus (GEO) database GSE22138 and GSE84976. Kaplan–Meier and Cox regression of univariate analyses were subjected to identify m7G-related regulators as prognostic genes.

**Result:**

A prognostic risk model comprising EIF4E2, NUDT16, SNUPN and WDR4 was established through Cox regression of LASSO. Evaluation of the model’s predictability for UVM patients’ prognosis by Receiver Operating Characteristic (ROC) curves in the training set, demonstrated excellent performance Area Under the Curve (AUC) > 0.75. The high-risk prognosis within the TCGA cohort exhibit a notable worse outcome. Additionally, an independent correlation between the risk score and overall survival (OS) among UVM patients were identified. External validation of this model was carried out using the validation sets (GSE22138 and GSE84976). Immune-related analysis revealed that patients with high score of m7G-related risk model exhibited elevated level of immune infiltration and immune checkpoint gene expression.

**Conclusion:**

We have developed a risk prediction model based on four m7G-related regulators, facilitating effective estimate UVM patients’ survival by clinicians. Our findings shed novel light on essential role of m7G-related regulators in UVM and suggest potential novel targets for the diagnosis, prognosis and therapy of UVM.

**Supplementary Information:**

The online version contains supplementary material available at 10.1186/s41065-024-00324-0.

## Introduction

Uveal melanoma originates from melanocytes in the eye with approximately 85% of tumor cases arising from choroid [1], it can also occur in the ciliary body or iris, and predominantly affects adults relative to children [2]. Up to 45% of uveal melanoma patients have metastasis within 15 years, that is prone to metastasize to liver (around 70%-90%) and with poor prognosis. Lack of effective treatment options on metastatic cases result in short survival time, commonly within one year [3, 4]. In recent years, numerous studies regarding the treatment of patients with metastatic UVM were focused on immunotherapy, such as immune checkpoint inhibitors [5, 6]. However, recent studies have found that certain inhibitors, such as anti-CTLA-4 antibody lpilimumab, have not achieved satisfactory results in the treatment of UVM patients [7]. Thus, the search for better biomarkers is extremely urgent, which are of profound importance in assessing patients' clinical outcomes and long-term prognosis. Epigenetic regulations, including lysine methylation and RNA modification, ubiquitously affect gene functions through the post-transcriptional modification, and these processes are vital to tumorigenesis [8]. It can potentially be applied as biomarker and of therapeutic targets for carcinomatosis [9]. RNA modification in epigenetics, especially RNA methylation, accumulating publications have been conducted on the topic in the last decade, excess of 100 types RNA modifications have been identified, the modification of m7G is one of the most universal forms of those, along with N6-methyladenosine (m6A) [10, 11]. At present, various lines of evidence show that the dysregulation of tRNA and its corresponding modification catalytic enzymes are involved in a variety of human cancers [12]. The most highly modified RNA molecule in cells is transfer RNA (tRNA) [13], which function as adapter molecules at post-transcription stage. The modification of m7G primarily occurs on tRNA within variable region located at position 46, produced by Methyltransferase for tRNA (m7G46) [14]. Methyltransferase methyltransferase-like protein-1(METTL1) and its’ partner WD repeat Domain 4 (WDR4), as catalytic enzymes of m7G methylation, that play a vital role in m7G methylation m7G [15]. Over the past few years, the role of m7G in cancer progression has been revealed in several studies [15–18], but the function and molecular mechanism of m7G in UVM is far from clear.

In present study, we collected data from the TCGA and GEO databases that including the clinical, mutation annotations and mRNA expression information of UVM patients. We constructed m7G-related risk model composed of four genes based on TCGA cohort of 80 UVM patients and further validated by two GEO cohort. Moreover, we analyzed the association with risk score based on the risk model and immune status in UVM patients. Our study provides a novel perspective on clinical diagnosis, prognosis and therapy of UVM patients.

## Materials and methods

### Dataset selection

The clinical, annotations of mutation and mRNA expression data of 80 UVM patients were collected from TCGA (https://portal.gdc.cancer.gov) as a training set of the m7G-related risk model. The clinical and mRNA expression data of GSE22138 (*N* = 63) and GSE84976 (*N* = 28) were selected from GEO (https://www.ncbi.nlm.nih.gov/geo), as the validation set by integration [19, 20]. Abstracts providing information about each dataset are presented in Table 1. We eliminated batch effects using the R packages "sva" and "limma". In the case of the same gene was probed with multiple probes, the expression of gene was measured by taking its average value. Using published reviews and Gene Set Enrichment Analysis (GSEA, http://www.gsea-msigdb.org) searches for "7-methylguanosine", 29 genes related to m7G were identified [14, 21, 22](Supplemental Table 1).

**Table 1:** Abstract of Information on datasets of TCGA, GSE22138 and GSE84976

Datasets	Component	Platform	Country	Version
TCGA	80 UVM tissue	Illumina HiSeq 2000	Bethesda, USA	2021
GSE22138	63 UVM tissue	GPL570 Affymetrix	Paris, France	2010
GSE84976	28 UVM tissue	GPL10558 Illumina	Leiden, Netherlands	2016

### Development and validation of the prognostic risk model

Genes of m7G-related regulators prominently associated with prognosis of OS were sifted by Kaplan–Meier and Cox regression of univariate (*p* < 0.05). Following that, regression analysis on the basis of the least absolute shrinkage and selection operator (LASSO), a popular penalization method, was utilized to process the prognostic genes obtained by applying the R package "glmnet" [23]. After the establishment of the prognostic risk model by LASSO, the result of the ten-fold cross-validation was used as the regularization parameter lambda (λ) of the risk model. We used the following calculation equation to measure the risk score of each sample: $Riskscore={\sum }_{i=1}^{i}\left(\text{Coefi}*\text{ExpGenei}\right)$.

ExpGene stood for the expression of each m7G-related gene, and coef stood for the coefficient of Cox regression for each gene and *n* as the number of genes included in the formula. Patients of UVM were separated into two cohorts, including high risk cohorts and low risk cohorts, based on their mid-value. ROC curves based on time-dependent were implemented using the R package "survminer" and "timeROC" to evaluate the precision of prediction [24]. For the purpose of comparing prognostic diversities between high-risk and low-risk cohorts, we executed Kaplan–Meier curves using the R package "survminer". R package "ggplot2" was used for Principal Component Analysis (PCA) analysis. In order to test if the risk model was a reliable independent prognostic factor based on OS, we enforced the analysis of the association with clinical characteristics and risk score by Cox regression, both univariate and multivariate.

### Comparison of cohorts at high and low risk on the basis of the m7G-related risk model

The DEGs were calculated by comparing cohorts at high and low risk. Subsequently, |log (FC)|> 1 and adjustments-*p*-value < 0.05 were the filter conditions to screen out prominent Differently Expressed Genes (DEGs). The analyses of Kyoto Encyclopedia of Genes and Genomes (KEGG, https://www.kegg.jp) and Gene Ontology (GO, http://geneontology.org) were implemented by R package "clusterProfiler". Immune-related pathways score and immune cells score were calculated for ssGSEA (Single Sample GSEA) by R package "GSEABase" and "GSVA". The proportion of immune matrix components in the tumor microenvironment (TME) was calculated by R package "estimate" to generate the immune score, matrix score, and Estimate score [25]. Thereafter, we calculated immune, matrix, and estimation scores for high-risk and low-risk cohorts, respectively.

### Statistical analyses

These analyses of statistics were enforced by the R software (R Foundation for Statistical Computing, 64-bit, R 4.1.2). Programming language Perl was used for bulk data processing (Perl 5.32.1). Statistical analyses of correlation and difference were carried out using R package "limma". Prominent differences were measured by the Wilcoxon rank-sum test in variables that were continuous between the two cohorts. The R packages "survminer" and "survival" were applied to implement the analysis of Kaplan–Meier. Statistical significance is considered at *p*-value < 0.05.

## Results

### Research process and clinical characteristics

**Fig. 1:**
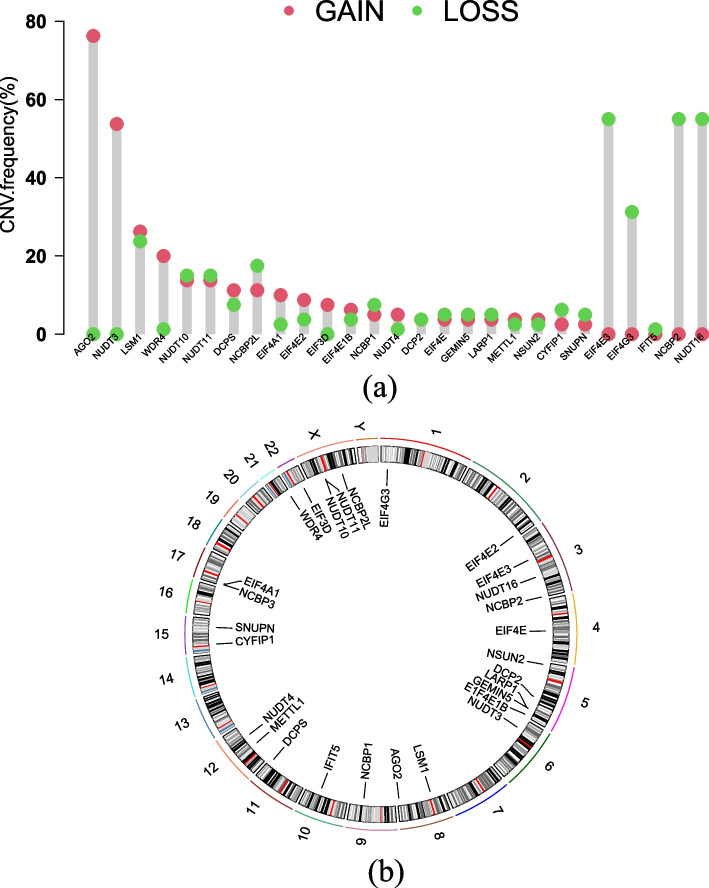
DNA copy number variation (CNV) frequency data of 29 genes associated with m7G and the corresponding positions of chromosomes. **a** Gene CNV frequency data of 29 m7g-related genes with UVM, gain is represented by the red dot and loss is represented by green dot. **b** The positions of 29 m7G-related genes on different chromosomes

Eighty UVM samples from TCGA database were selected as the training set, and 91 samples of GSE22138 and GSE84976 obtained from GEO database were chosen for validation set after data integration with R package "sva" to remove batch effect. A summary of clinical parameters is provided in Table 2. Next, using the DNA copy number variation (CNV) frequency data of UVM, we analyzed and visualized 29 m7G-related genes (Fig. 1a). The frequency of gain of AGO2 and NUDT3 was significantly higher than its loss, while the results of EIF4E3, EIF4G3, NCBP2 and NUDT16 were contrary. The positions of 29 m7G-related genes on different chromosomes were illustrated in Fig. 1b. Notably, among the first three genes in which the frequency of loss was significantly greater than gain, they were located on chromosome 3.

**Table 2:** Summary of UVM patients’ clinical information

Clinical characteristics		TCGA(*N* = 80)	GSE22138(*N* = 63)	GSE84976(*N* = 28)
Age	< 60	36	28	12
	≥ 60	44	35	16
Gender	Male	45	39	NA
	Female	35	24	NA
Statue	Alive	57	28	12
	Dead	23	35	13
Stage	Stage II	39	NA	NA
	Stage III	37	NA	NA
	Stage IV	4	NA	NA
Tumor diameter(mm)	< 12	6	11	NA
	≥ 12	73	42	NA
Tumor thickness(mm)	< 60	36	28	12
	≥ 60	44	35	16
Extrascleral extension	Yes	7	5	NA
	No	68	48	NA
	Unknow	5	10	NA

### The prognostic risk model is developed and validated on the basis of m7G-related regulators

**Fig. 2:**
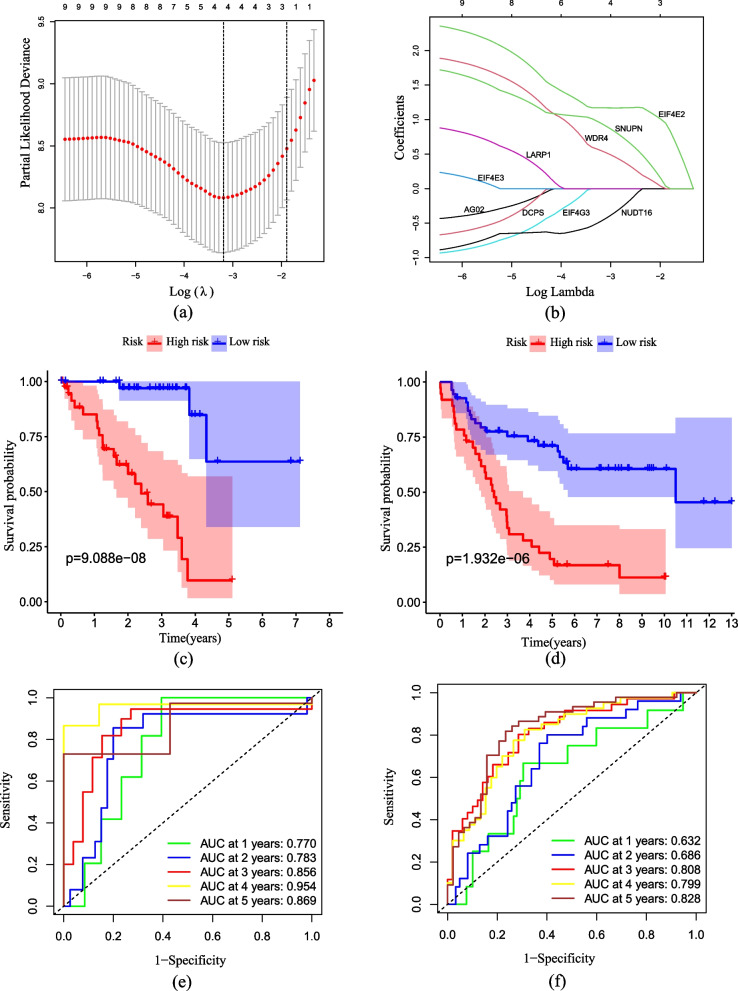
Developing and validating the prognostic risk model on the basis of m7G-related regulators. **a** LASSO coefficient profiles of the four genes which belong to the m7G-related risk model. **b**The penalty regularization parameter lambda (λ) was determined by the result of the ten-fold cross-validation. **c**, **d** The curves of Kaplan–Meier analysis for TCGA cohort (**c**) and GEO cohort (**d**). **e**, **f** ROC analysis for TCGA cohort (**e**) and GEO cohort (**f**)

Following extracting the expression data of 29 m7G-related genes from 80 UVM samples from TCGA, we carried out Cox regression of Kaplan–Meier and univariate analyses. Nine m7G-related genes were identified, and they were remarkably associated with prognosis in terms of OS. Integrating the nine genes into Cox regression of LASSO, the LASSO coefficient curve consisting of four m7G-related prognostic genes was obtained, with the minimum parameter serve as the penalty regularization parameter lambda (λmin = 0.0411, Fig. 2a-b and Supplemental Table 1–3). The LASSO Cox regression model is: Risk score = (1.170190103 × mRNA expression of EIF4E2) + (-0.470940387 × mRNA expression of NUDT16) + (0.55003035 × mRNA expression of SNUPN) + (0.945486337 × mRNA expression of WDR4) (Table 3). Using the mid-value of risk score as the cut-off, UVM patients were categorized into high and low risk cohorts.

**Table 3:** LASSO identified four genes associated with m7G

Gene	Coef
EIF4E2	1.170190103
NUDT16	-0.470940387
SNUPN	0.55003035
WDR4	0.945486337

According to the Kaplan–Meier curve (*p* < 0.001), the cohort at high risk in Fig. 2c exhibited poor performance. Subsequently, the risk model’s predictive ability the OS prognosis by calculating 1, 3 and 5-years AUC values (AUC at 1, 3 and 5-years was 0.770, 0.856, and 0.869, respectively, as shown in Fig. 2e). The risk model demonstrated reliable long-term prognostication. Consistent with the training set, the Kaplan–Meier curve for the validation set showed poor prognosis in the high risk cohort (*p* < 0.001, Fig. 2d), Additionally, Fig. 2f was generated through ROC analysis, displaying AUC value at 1, 3 and 5-years (0.632, 0.808 and 0.828, respectively). These findings further support the predictability of the risk model for the prognosis of OS.

**Fig. 3:**
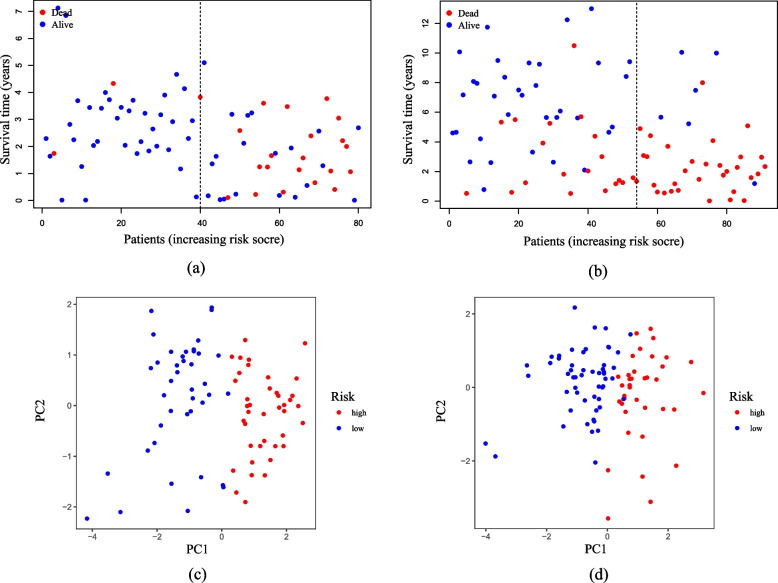
The distribution of the risk plot and PCA. **a**, **b** Analyses of the distribution based on risk score and survival status for TCGA cohort (**a**) and GEO cohort (**b**). **c**, **d** PCA plot of TCGA cohort (**c**) and GEO cohort(**d**)

To comprehensively assess the risk model, we calculated and visualized both the risk plot and PCA distribution for the training and validation sets, respectively (Fig. 3a-d). UVM Patients from the cohort at high risk exhibited a poor prognosis compared to those from low risk cohort. Furthermore, PCA analysis illustrated that the risk model effectively distinguish between the high and low risk cohorts. Therefore, the risk model, based on four genes related to m7G-related regulators was successfully established to assess the prognosis of UVM patients.

### Correlation between clinical characteristics and risk score

**Fig. 4:**
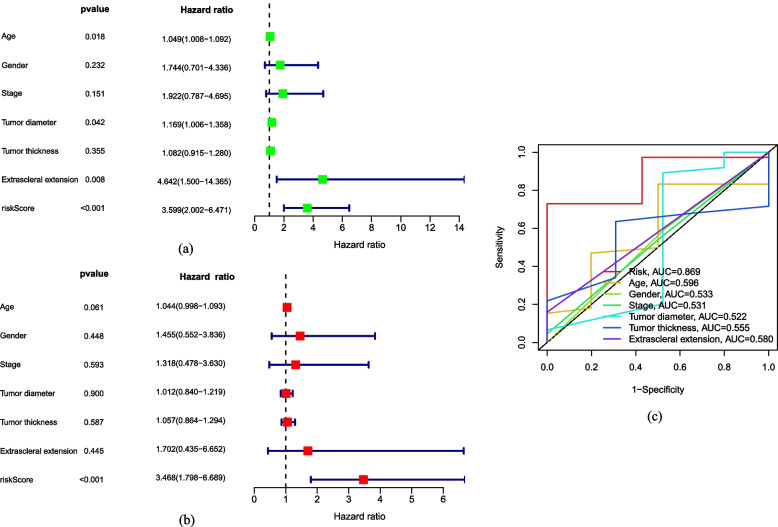
Correlation between clinical characteristics and risk score. The Cox regression of univariate (**a**) and multivariate (**b**) between clinical characteristics and risk score. **c** ROC analysis on risk score and clinical characteristics

To rigorously evaluate the risk model as robust independent indicator of OS prognosis in UVM patients, both univariate and multivariate Cox regression analyses were enforced between the high-risk and the low-risk cohorts. We integrated clinical information from 80 UVM samples in TCGA, including age, gender, stage, tumor diameter, tumor thickness and extrascleral extension. Univariate Cox regression results revealed that age, stage, tumor diameter, extrascleral extension and risk score were prominently independent variables of OS prognosis in UVM patients (*p* < 0.05, Fig. 4a). In multivariate Cox regression analysis, risk score remained an independent variable of OS prognosis in UVM patients (*p* < 0.05, Fig. 4b). This underscores the validity of the risk model based on m7G-related regulators in predicting UVM prognosis. Additionally, we performed a ROC analysis based on time-dependent for both risk score and clinical characteristics. As depicted in Fig. 4c, the AUC of risk score was 0.869, surpassing the AUC that based on clinical characteristics. This result strongly indicates that risk score provide a more accurate prediction in prognosis compared to clinical characteristics.

### Gene set enrichment analysis

**Fig. 5:**
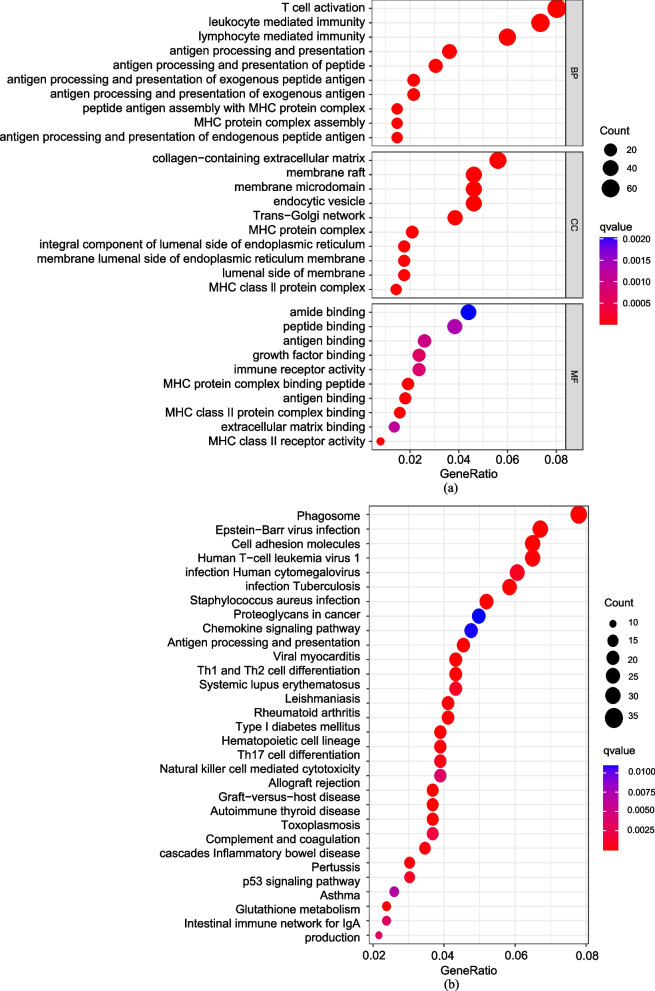
KEGG and GO enrichment analysis. The bubble belongs to enrichment analyses of KEGG (**a**) and GO (**b**) in DEGs for TCGA cohort

To further elucidate differences in pathway and gene function between high-risk and low-risk cohorts categorized based on risk model, we identified the DEGs of TCGA cohort using condition (*p* < 0.05 and |logFC|> 1). Subsequently, we performed KEGG and GO based on these DEGs. In KEGG pathway enrichment analysis, the result revealed that the risk model was closely correlated with phagosome, infection and cell adhesion molecules (Fig. 5a). In GO function enrichment analysis, T cell activation exhibits the most significant enrichment, followed by leukocyte-mediated immunity and lymphocyte mediated immunity, among others. The risk model based on m7G-related regulators exhibited a strong association with T cell function and immune (Fig. 5b). These findings suggest that the identified risk model might play a crucial role in modulating T cell activities and immune responses.

### Correlation between somatic mutations and risk score

**Fig. 6:**
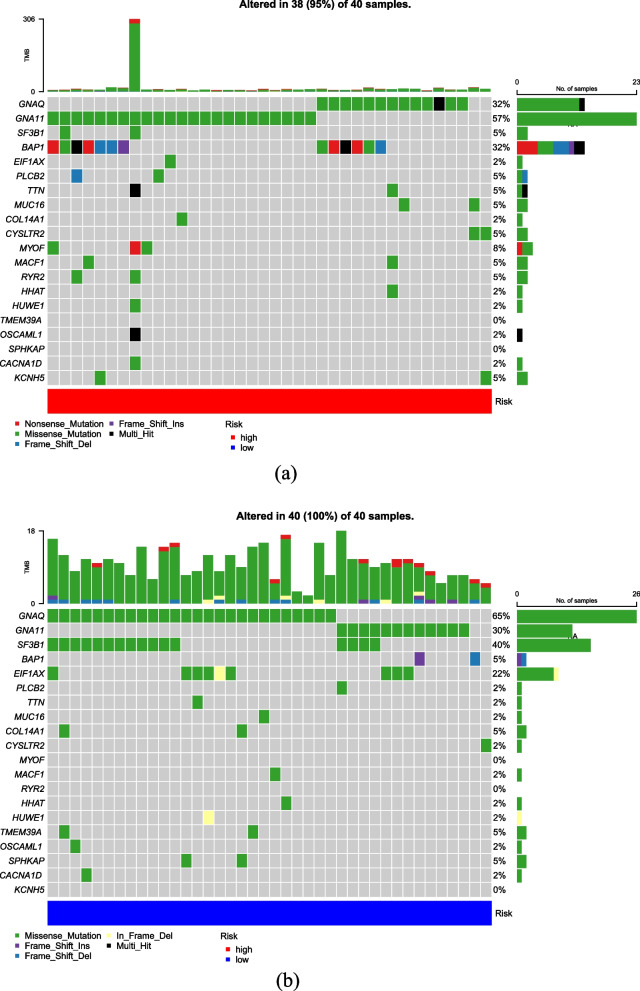
Mutation map of cohorts at high and low risk cohorts. Mutation map of the top twenty mutated genes for (**a**) high-risk cohort and (**b**) low-risk cohort

In the high and low risk cohorts, R package "maftools" was employed to analyze the distribution of mutational differences between m7G-related genes. The top twenty genes with the highest mutation rates were selected and visualized in Fig. 6a-b. The waterfall plots depicted that the most significant pre-mutation gene in low risk cohort, was GNA11, while, in the low risk cohort was GNAQ. These findings suggest distinct mutational landscapes in m7G-related genes between the two risk groups.

### Correlation between immune characteristics and risk score

**Fig. 7:**
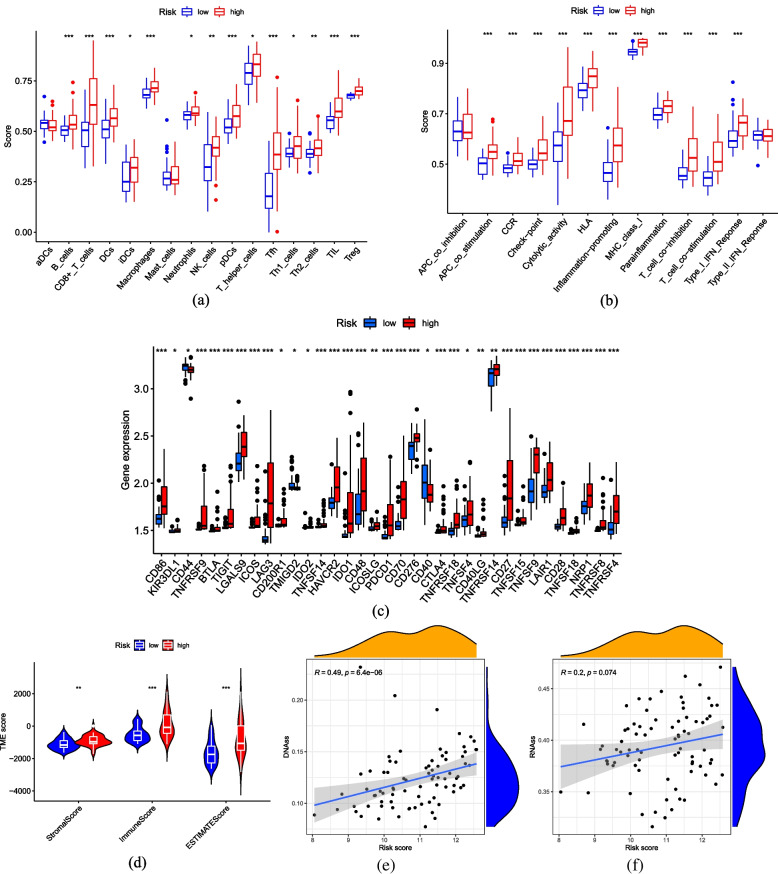
Correlation between immune characteristics and risk score. The immune cell subpopulations (**a**) and functions (**b**) between cohorts at high and low risk based on the ssGSEA. **c** The mRNA expression of each gene related to immune checkpoints of cohorts at high and low risk. **d** The violin figure of immune, stromal and ESTIMATE scores for cohorts at high and low risk. **e** DNAss. **f** RNAss

The risk model we developed may be closely related to immunity in gene set enrichment analysis. Therefore, we analyzed the differences in immune characteristics between high-risk and low-risk cohorts from the TCGA-UVM. In our investigation using ssGSEA, we have found that most immune cell subpopulations and functions were significantly enhanced in the high-risk cohort (*p* < 0.05, Fig. 7a,b), it suggests that the UVM high-risk cohort exhibits over expression of immune-related functions. Next, we compared the mRNA expression of genes related to immune checkpoints between the two cohorts. Immune checkpoint-related genes exhibited higher expression levels in high-risk cohorts, as depicted in (Fig. 7c). Using R package "estimate" analysis to reflect the immune microenvironment landscape of tumor, we found that immune, stromal and estimate scores of high-risk cohort were significantly enhanced (*p* < 0.001), each correlation coefficient was greater than 0.34 in absolute value (Fig. 7d). In addition to immune microenvironment, tumor stemness scores, encompassing DNA methylation profiles and RNA stemness scores, also played a pivotal role in tumor progression. Consistent with the results of immune microenvironment, DNA methylation profiles showed a strikingly positive relationship with UVM risk score (*R* = 0.49, *p* < 0.05, Fig. 7e), while RNA stemness score did not exhibit a remarkable significant positive correlation in our results (*R* = 0.2, *p* = 0.074, Fig. 7f). These findings provide further support for our earlier findings that risk score based on m7G-related risk models is closely related to immune activation.

## Discussion

M7G, N7-methylguanosine, represents a one form of RNA modification. This modification is known to enhance mRNA stability and occurs prior to changes at the protein level, suggesting its potential advantage in predicting disease progression. Focusing solely on modifications to individual genes may limit the accuracy and comprehensiveness needed to predict the course of a disease. In this study, nine m7G-related prognostic regulators were identified by Kaplan–Meier and univariate Cox analyses in the TCGA cohort. The risk model for UVM prognosis was established using LASSO Cox regression, which consisted of four m7G-related genes (EIF4E2, NUDT16, SNUPN and WDR4). To evaluate the efficacy of m7G-related risk model, we stratified the TCGA cohort was into high-risk and low-risk cohorts with the mid-value approach. We then carried out Kaplan–Meier curves and ROC analysis between these two cohorts. Strikingly statistical differences show high-risk cohort exhibited worse prognosis compared to those in low-risk counterparts. The AUC at 1, 3 and 5-years were 0.770, 0.856, and 0.869, respectively, suggesting strong predictive capability on prognosis of OS of UVM. Furthermore, we conducted both univariate and multivariate Cox regression to assess the correlation between risk score and major clinical characteristics. Our results confirmed that our four genes m7G-related risk model was reliably independent of other variables that predicted UVM outcomes. To validate our findings, we applied in GEO database and visualized the risk plot and PCA distributions. the AUC at 1, 3 and 5-years was 0.632, 0.808 and 0.828, respectively. This validation further demonstrated that the m7G-related risk model was a reliably independent indicator of OS prognosis.

Among the four prognostic genes of risk model, WDR4 plays a non-catalytic role in m7G methyltransferase complex. The complex formed by METTL1 and WDR4 catalyzes the methylation at position 46 of tRNA, that primarily stabilizing WDR4 structure [26, 27]. It had been demonstrated experimentally that METTL1/WDR4 can regulate the development of cancer through tRNA modification of m7G methylation in intrahepatic cholangiocarcinoma, lung cancer and nasopharyngeal carcinoma [15–18]. *Jieyi* *Ma *et al*.* observed a striking deceleration in lung cancer progression by knocking down METTL1/WDR4 [17]. Eukaryotic translation initiation factor 4E family member 2 (EIF4E2), belongs to eukaryotic promoter factor, is a cap-binding protein that binds to 7-methylguanosine-containing mRNA caps during translation initiation [28]. Nudix hydrolase 16 (NUDT16) is a nuclear RNA decapping protein, *Taylor MJ *et al*.* reported that NUDT16 is capable of binding and hydrolyzing the m7G cap from the 5'-end of RNA in vitro analysis of the insect ortholog [29]. Another study of *Shaohong* *Peng *et al*.* found that NUDT16 silencing leas to apoptosis and DNA damage [30]. Snurportin 1 (SNUPN), an adaptor protein, is a nuclear import receptor specifically associated with spliceosomal snRNPs U1, U2, U4 and U5. Moreover, SNUPN encoded proteins interact specifically with m3G-Cap and m7G-Cap [31]. In addition to NUDT16 being a protective factor, the other three genes (WDR4, EIF4E2, and SNUPN) are risk factors that accelerate tumor progression. Our results regarding m7G modification in the above four genes were consistent with the previous studies that have been reported to affected tumor progression.

Multiple lines of evidence have demonstrated RNA methylation closely correlated with tumor immunity, highlighting its crucial clinical implications. In our study, KEGG pathway enrichment analysis revealed that the main pathways affected are phagosome, infection and cell adhesion molecules, Meanwhile, the primary outcomes of GO functional enrichment analysis included T cell activation top the BP section, leukocyte-mediated immunity and lymphocyte-mediated immunity, indicating a close association between immune response and the risk score calculated by the m7G-related risk model. Subsequently, the ssGSEA and TME analyses were carried out and both indicating a higher level of immunoreaction in the high-risk cohort. It’s well- known that to prevent damage to non-regenerative tissues such as neural retina by immunoreactivity, normal organism has the immune privilege to their eyeballs [32]. The Kaplan–Meier curve of Fig. 3c, we observed strikingly poor prognosis in the high-risk cohort. Combined with the previous results, we hypothesize that the dysregulation of m7G related genes in UVM patients is closely related to the enhancement of ocular immunoreaction, especially T cells and leukocytes. This hyperactive immune response compromises the eyeball's local immunity, contributing to worse outcomes in UVM patients. based, The comprehensive AUC value based on our 4 genes risk model of m7G-related regulators is 0.869, precisely predicting the prognosis of UVM patients In contrast to the significant strides made in cutaneous melanoma treatment the impact of immune checkpoint inhibitors on UVM is constrained, with the evidence of drug resistance [33]. UVM patients exhibiting high scores of m7G-related risk model displayed increasing abundant of immune infiltration, notably characterized by a general up-regulation of immune checkpoint related genes. This suggested that UVM patients with high score exhibit higher sensitivity to immune checkpoint inhibitors. Clinical application of the m7G-related risk model can help doctors timely identifying UVM patients suitable for immunotherapy. In conclusion, UVM patients exhibiting high risk score stratified by the m7G-related risk model are more likely to respond positively to immunotherapy, which has strikingly implication for application of immune checkpoint inhibitors in clinical settings. However, it's crucial to acknowledge the limitations in our research such as limited samples incorporated. This model may necessitate more prospective data from multiple center studies.

## Conclusions

In this study, we utilized data from TCGA and GEO database to formulate a prognostic risk model comprising four m7G-related genes, suggesting that m7G based modification may shed a novel perspective on diagnosis, prognosis, survival and therapeutic targets correlated with UVM.

### Supplementary Information

Supplementary Material 1.

Supplementary Material 2.

Supplementary Material 3.

## Data Availability

The original data involved in this work comes from the website: https://portal.gdc.cancer.gov; https://www.ncbi.nlm.nih.gov/geo; http://www.gsea-msigdb.org. The code and results used to support the findings of this study are available from the corresponding author.

## Abbreviations

UVM: Uveal melanoma
m7G: N7-methylguanosine
TCGA: The Cancer Genome Atlas
GEO: Gene Expression Omnibus
ROC: Receiver Operating Characteristic
AUC: Area Under the Curve
m6A: N6-methyladenosine
METTL1: Methyltransferase methyltransferase-like protein-1
WDR4: WD repeat Domain 4
GSEA: Gene Set Enrichment Analysis
LASSO: Least absolute shrinkage and selection operator
PCA: Principal Component Analysis
DEGs: Differently Expressed Genes
TME: Tumor microenvironment
ssGSEA: Single Sample GSEA
CNV: DNA copy number variation
EIF4E2: Eukaryotic translation initiation factor 4E family member 2
