# Correction: Development of a prognostic risk model of uveal melanoma based on N7-methylguanosine-related regulators

**DOI:** 10.1186/s41065-024-00326-y

**Published:** 2024-07-24

**Authors:** Pingfan Wu, Qian Zhang, Peng Zhong, Li Chai, Qiong Luo, Chengyou Jia

**Affiliations:** 1 Department of Nuclear Medicine, Shanghai Tenth People’s Hospital, Tongji University School of Medicine, Shanghai, 200072 China; 2 Institute of Nuclear Medicine, Tongji University School of Medicine, Shanghai, 200072 China; 3 Department of Plastic and Aesthetic Surgery, The Second Affiliated Hospital of Soochow University, Suzhou, 215004 China

**Correction:. Hereditas 161**,** 22 (2024)**

10.1186/s41065-024-00324-0

Following publication of the original article [1], the author reported that the affiliation numbers were incorrectly assigned to their names and want to update from:

Pingfan Wu^1,2†^, Qian Zhang^3†^, Peng Zhong^1^, Li Chai^3^, Qiong Luo^3^ and Chengyou Jia^1*^.

Into

Pingfan Wu^1,3†^, Qian Zhang^1,2†^, Peng Zhong^1^, Li Chai^1,2^, Qiong Luo^1,2^ and Chengyou Jia^1 2*^.

This has been updated above and the original article [1] has been corrected.
